# Using the Virtual Reality-Cognitive Rehabilitation Approach to Improve Contextual Processing in Children with Autism

**DOI:** 10.1155/2013/716890

**Published:** 2013-11-13

**Authors:** Michelle Wang, Denise Reid

**Affiliations:** ^1^Office of Undergraduate Medical Education, Queen's University, 80 Barrie Street, Kingston, ON, Canada K7L 3N6; ^2^Virtual Reality and Neurorehabilitation Laboratory, University of Toronto, 160-500 University Avenue, Toronto, ON, Canada M5G 1V7

## Abstract

*Background*. This pilot study investigated the efficacy of a novel virtual reality-cognitive rehabilitation (VR-CR) intervention to improve *contextual processing of objects* in children with autism. Previous research supports that children with autism show deficits in contextual processing, as well as deficits in its elementary components: abstraction and cognitive flexibility. *Methods*. Four children with autism participated in a multiple-baseline, single-subject study. The children were taught how to *see objects in context* by reinforcing attention to pivotal contextual information. *Results*. All children demonstrated statistically significant improvements in contextual processing and cognitive flexibility. Mixed results were found on the control test and changes in context-related behaviours. *Conclusions*. Larger-scale studies are warranted to determine the effectiveness and usability in comprehensive educational programs.

## 1. Introduction

Autism or autism spectrum disorders (ASD) refer to a group of neurodevelopmental disorders that are characterized, in differing degrees, by core deficits in social and communication skills, as well as distinct abnormal behaviours [1]. The prevalence of autism in children is approaching 1%, or approximately 1 in 110 children [2]. Although autism can be diagnosed as young as 18 months of age [3], the symptoms of this disorder last throughout an individual's lifetime.

An innovative combination of traditional cognitive rehabilitation and virtual reality technology offers an interactive, cognitive approach to autism intervention [4]. This emerging framework focuses on remediating underlying cognitive impairments of disorders and uses virtual reality technology to maintain a high level of engagement and attention from the children. The current study used this approach to improve a specific cognitive impairment in children with autism: *contextual processing of objects*.

### 1.1. The VR-CR Approach

The virtual reality-cognitive rehabilitation (VR-CR) framework was first proposed by Rizzo and Buckwater [4] for children with attention-deficit hyperactivity disorder (ADHD). Traditional cognitive rehabilitation employs specific exercises designed to improve cognitive functions through repetitive training exercises that specifically target the impaired component processes. Intense repetition of specific exercises is necessary to reorganize the brain in a particular area; however, it places immense demands on both the child and the instructor [5]. This presents a greater problem when extending the cognitive rehabilitation approach to children with autism, as they are particularly difficult to engage. Virtual reality makes cognitive programs accessible to children with autism through its capacity to maintain their attention, provide structured and individualized activities, and address their weaknesses while building on their strengths [6]. 

Virtual Reality is defined as a simulation of the real world using computer graphics [7]. The defining features of a VR program or application include *interaction* and *immersion*. Human-computer interaction is achieved through multiple sensory channels that allow children to explore virtual environments through sight, sound, and touch [7]. Immersion is considered the degree to which the child feels engrossed or enveloped within the virtual environment [8]. 

A variety of display devices offer differing degrees of immersion and interactivity. The current study employs a two-dimensional flat screen projection system. This system has motion-capture capabilities, where a tracking camera is able to capture and project a child's image and motions on-screen in real-time. Being able to see oneself within the virtual environment may contribute to a greater sense of engagement and motivation during the task. 

Although VR systems have not yet targeted cognitive impairments in children with autism, these systems have been successful in improving specific behaviours such as following directions [9], crossing the street [10], finding a seat on the bus [11], ordering coffee in a café [11], and exiting a building during a fire alarm [12]. Overall, VR systems provide the instructor with a balance between flexibility and control. In the context of a cognitive rehabilitation program, VR systems allow repetitive exercises to be presented in a motivating, engaging, and naturally reinforcing way. A more thorough description of using the VR-CR approach with children with autism is provided by Wang and Reid [13]. The current study employed the combined VR-CR approach to address and improve a specific cognitive impairment in children with autism, an impairment in *contextual processing of objects*.

### 1.2. Contextual Processing of Objects

Contextual processing of objects is defined as the ability to determine an object's meaning or relevance in a particular context [14]. Objects are inherently multidimensional; each encompasses simple, concrete qualities such as colour, as well as more complex, abstract dimensions such as roles and spatial arrangements [14]. To determine an object's meaning or significance in a multiobject context, one must take into consideration the relationships that make a target object relevant within a context, as well as adapt flexibly to changing contexts. The three major types of dimensional relationships between objects and their contexts are perceptual (colour, shape, size), spatial (location), and functional (role or use) [14, 15]. The relevant relationships between an object and its context are, in large part, determined by top-down attentional control that is formed from a person's expectations and stored mental representations of that object [14]. 

Contextual processing can be deconstructed into two executive functions: *abstraction*, the ability to identify the context by extracting the relevant qualities and relationships within the environment, and *cognitive flexibility*, the ability to switch between multiple mental representations of a single object in response to changing contextual factors. 

### 1.3. Impaired Contextual Processing in Children with Autism

Categorization tasks are typically used to assess contextual processing. These tasks require the child to *abstract* a particular object dimension by assigning objects to groups based on internally generated or spontaneous sorting criteria. The categorization task evaluates cognitive flexibility when the child is asked to *change* sorting criteria. Changing criteria requires the child to make a mental switch between multiple object representations. 

Ropar and Peebles [16] investigated the spontaneous sorting preferences of children with autism and provided initial evidence that children with autism have difficulty abstracting higher-level object categories (e.g., types of sports, games). Klinger and Dawson [17] reported that during rule-based category learning tasks, (the children with autism performed as well as typical controls). However, the performance of the children with autism significantly decreased during prototype-based category learning tasks. As prototype creation involves abstracting and integrating relevant information from members of a category, these results further the notion that children with autism fail to integrate information at an abstract level.

Alternatives to the categorization test include two novel assessments created by Jolliffe and Baron-Cohen [18] to evaluate contextual abstraction in adults with autism. The first test is the Object Integration test, which requires the participant to identify which of five objects is incongruent by establishing a common context between the displayed objects. The commonalities between the objects include either spatial or functional relationships. The second test is the Scenic Integration test in which a complex visual scene is presented where one object is incongruent. The participants are required to identify the incongruent object based on the established context. Performance results on these tests showed that adults with autism made significantly more errors and took more time to complete both tests as compared to typical controls. The authors concluded that the individuals with autism failed to use context to complete the tests [18]. Overall, the Object Integration test and Scenic Integration test provide alternatives to evaluate abstraction abilities in individuals with autism.

Research has also shown pervasive impairments in cognitive flexibility in individuals with autism. The Wisconsin Card Sorting Task (WCST) [19, 20] is a derivative of the simple categorization task; however, in addition to sorting cards based on particular dimensions (e.g., colour, shape, size), the child must *change* sorting criterion according to feedback received by the experimenter. The degree of perseveration, or failure to switch sorting criteria, is an effective indicator of cognitive flexibility. A high degree of perseveration on the original and computerized WCST in children and adults with autism has been well-replicated [21–24]. Although the WCST test does not evaluate cognitive flexibility beyond simple, concrete object dimensions, it does provide evidence that even at this simple categorization level, cognitive flexibility, in the realm of object processing, is impaired.

In summary, the evidence strongly supports the existence of contextual processing impairments in children with autism. These studies demonstrate impairments in the ability to abstract relevant contextual information and the ability to flexibly switch mental representations as a function of changing contexts. The purpose of the current pilot study was to evaluate a virtual reality-cognitive rehabilitation (VR-CR) intervention to improve contextual processing of objects in children with autism.

### 1.4. Objectives

The objectives of the pilot study were

1. to explore the efficacy of a novel VR-CR intervention for improving contextual processing of objects, abstraction, and cognitive flexibility in four children with autism;
2. to explore parent perceptions of behavioural changes that may have occurred during the intervention.

## 2. Material and Methods

### 2.1. Experimental Design

The current study used a single-subject design with nonconcurrent multiple baselines across subjects. Four children were enrolled in the pilot study. Each child was studied over four to six weeks. The study consisted of a baseline phase, training phase, and a two-week follow-up session, with outcomes assessed throughout. Prior to enrolment, the children were randomized to baseline lengths of 3, 4, 5, and 6 sessions.

### 2.2. Participants

Ethical approval for the current study was obtained from the local hospital and university research ethics boards. The inclusion criteria were as follows: (1) diagnosis of an autism spectrum disorder (ASD) by a pediatric neurologist, pediatrician, clinical psychologist, or psychiatrist (copy of child's diagnostic report required); (2) chronological age between 5 and 10 years old; (3) autism severity classification of mild to moderate (Childhood Autism Rating Scale score of 30–36); and (4) average or above-average nonverbal IQ (Leiter Brief-IQ score of 85 or greater). A summary of the participants' demographic information is provided in Table 1. The children's names have been changed to protect confidentiality.

### 2.3. Setting and Equipment

The study was conducted in a quiet room in the children's homes. The virtual reality training programs and virtual reality tests were displayed on a 15′′ Acer TravelMate 8204 laptop computer. Motion-capture technology was incorporated using a tracking webcam (Logitech QuickCam Pro 9000) to capture and project the child's image and movements into the virtual environment. All software programs were programmed using Flash 8 with the programming language Actionscript 2.0. The programs were run using Macromedia Flash Player. The VR program required the children to drag virtual objects to specific locations on the laptop screen.

### 2.4. Outcome Measures

#### 2.4.1. Virtual Reality Test of Contextual Processing of Objects (VR Test)

The VR test is an adaptation of the Object Integration test by Jolliffe and Baron-Cohen [18], which evaluates contextual processing of objects in children between ages 5 and 10 years. To complete the task, the child is required to make a similarity judgment between a movable target object and a multiobject context displayed on the screen. The context contains three objects that highlight a particular object dimension: perceptual (e.g., colour, shape), spatial (e.g., kitchen, bathroom), or functional (e.g., objects used to cut). The purpose of the task is to determine if the target object is meaningful in the given context (i.e., if it shared the same relevant dimension). 

There are two equivalent versions of the VR test. Each version is composed of 18 test items (object-context pairs). Six pairs are matched based on perceptual relationships, six pairs are matched based on the spatial dimension, and six pairs are matched based on functional characteristics. Half of the pairs are matched correctly, and half are matched incorrectly. The 18 test items are randomized differently for each test version. The VR test was administered during each session. The software program and the researcher independently record the correct and incorrect responses for each test item. The child does not receive feedback for his or her responses on the VR test, thus minimizing the occurrence of learning effects as a result of repeated administration. 

#### 2.4.2. Modified Version of the Flexible Item Selection Task (FIST-m)

The Flexible Item Selection Task (FIST) [27] was used to assess executive functioning. On each test item, three objects are shown (e.g., red fish, blue fish, and red telephone). On the first part of the task (Selection 1), the child is asked to point to two objects that “go together.” These two items match on one relevant dimension (e.g., common object: red fish and blue fish). Then the child is asked to point to a different pair of objects that “go together” (Selection 2). This new pair matches on a different dimension (e.g., common colour: red fish and red telephone). The common item in both pairs is the “pivot item” (e.g., red fish). Selection 1 involves the ability to internally abstract a relevant dimension to identify the pairs. Although Selection 2 also requires abstraction, it is a good measure of cognitive flexibility, as the child must be able to switch between different mental representations of the pivot object. 

As the original FIST was developed for preschoolers, the items of the FIST-m have been adapted for the older 5-to-10 age group. Similar to the original, the FIST-m comprises 12 test items in total and includes items from the original test. The FIST-m was administered at prebaseline, posttraining, and follow-up sessions.

#### 2.4.3. Attention Sustained Subtest [26]

The Attention Sustained Subtest from the Leiter International Performance Scale-Revised is a cancellation test which measures prolonged visual attention, visual scanning, and visuomotor inhibition. It consists of four separate test trials that require the child to colour a target shape or pattern (e.g., a square) within a complex array of different shapes. None of the cognitive constructs evaluated by this subtest were explicitly taught in this study; thus it was used as a control test. The Attention Sustained Subtest was administered at prebaseline, posttraining, and follow-up sessions.

#### 2.4.4. Final Feedback Questionnaire

The Final Feedback Questionnaire was developed to provide insight into parental perceptions of behaviour changes in their children. The behaviours on the form are those associated with contextual processing and included seven general categories of behaviour, in particular, behaviour in public contexts, language and communication in social contexts, and flexible use of objects. These categories of behaviours were chosen based on reported correlations between these behaviours and cognitive impairments [28–31]. The specific examples for each category were taken from the Vineland Adaptive Behaviour Scale (VABS) [32]. The Final Feedback Questionnaire was administered once at the end of the study.

### 2.5. Procedure

#### 2.5.1. Baseline Phase

Before initial testing, the children were provided with prebaseline training to ensure that they understood the instructions associated with the VR test. Using simplified pretest items (e.g., shapes), the child's responses were modelled, prompted, and reinforced during this pretraining task.

#### 2.5.2. Training Phase

The training program was comprised of three discrete lessons, with one lesson taught per session. Each lesson focused on one class of object characteristics: perceptual, spatial, or functional. The goal of each lesson was to teach the child to flexibly attend to object dimensions of that particular class. The lessons were taught in the order as specified above, and mastery on the preceding lesson was required to advance to the next. Each training session involved a 10-minute teaching protocol during which the same researcher provided each child with one-on-one instruction within the virtual environment. The training sessions were designed to support the child's understanding and performance on the task. Verbal instructions, modelling, prompting, repetition, and reinforcement were the teaching strategies that were incorporated.

The virtual reality program presented each set of 10 training items in a predictable sequence. First, the multiobject context screen was shown (e.g., three objects displayed on a table), then the target object appeared. The motion-capture virtual technology allowed the child to see him/herself on-screen and to indicate responses through gestures. The child was able to “grab and drag” the target object across the screen. Dragging the target object towards the other items indicated a positive “match,” while dragging the target to the garbage bin indicated a negative “nonmatch.” Visual reinforcement was built into the training program, with correct responses rewarded with a happy face, while incorrect responses were discouraged with a sad face. There was no overlap in the items used on the VR test and those used in the training sessions. 

**Figure 1:**
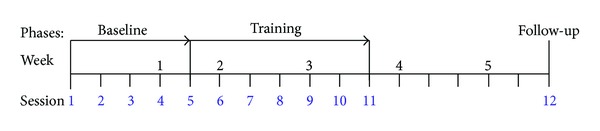
Overall study design for a hypothetical participant with a 5-session baseline phase, 6-session training phase, and 2-week follow-up session.

After completing each lesson, the VR test was administered. After each test administration, the VR test was analyzed according to its separate components: perceptual, spatial, and functional items. A score of 80% or above was considered mastery. For example, if the child scored over 80% on the VR test perceptual items after Lesson 1, she/he would progress to Lesson 2. A child who failed to achieve 80% on the VR test perceptual items after Lesson 1 would repeat that lesson. The training phase was ended when the child achieved over 80% in all three components of the VR test (see Figure 1).

## 3. Results

### 3.1. VR Test

**Figure 2:**
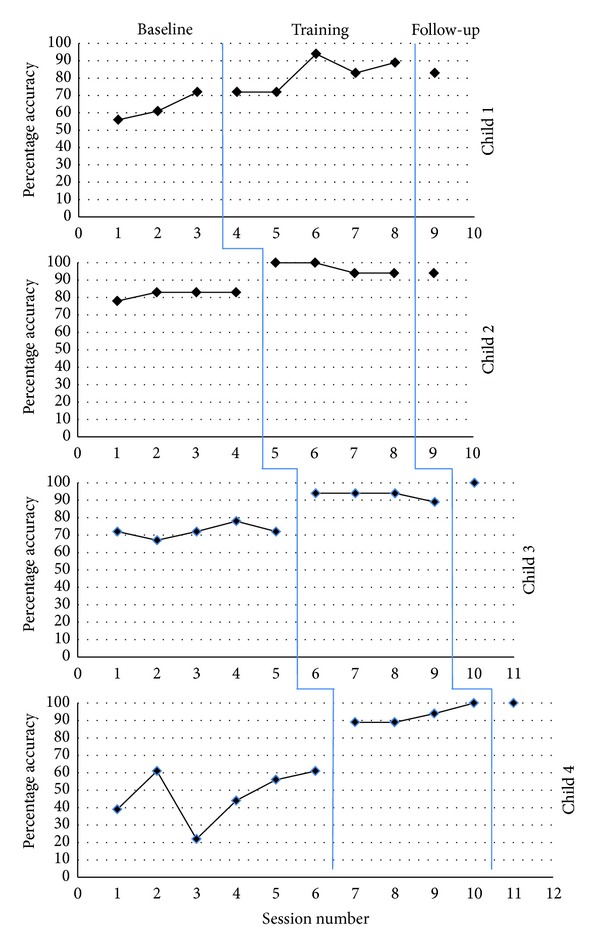
Percentage accuracy on the VR test demonstrated by each child across all phases of the study.

The VR test percentage accuracy scores are presented for each child across each session (Figure 2). The average baseline scores ranged from 47% (Child 4) to 83% (Child 2). Child 2, Child 3, and Child 4 showed marked improvements after the first training session, which were maintained throughout the training phase and at follow-up. Child 1 showed significant improvements after the third training session and maintained this at follow-up.

The Percentage Nonoverlapping Data statistic was used to analyze the data [33]. Child 2, Child 3 and Child 4 all demonstrated 100% nonoverlapping data. Child 1's performance data showed 60% nonoverlapping data. 

Overall, the results demonstrate improvements in contextual processing ability from baseline to treatment for each child, with average increases from 15% (Child 2) to 46% (Child 4). All children maintained a high level of performance at the two-week follow-up assessment.

### 3.2. FIST-m

Percentage accuracy scores were calculated for pretraining and posttraining administrations of the FIST-m.  Table 2 shows these data for Selection 1 and Selection 2 separately.

All four children displayed ceiling or close to ceiling scores on Selection 1 (abstraction) scores at pretraining. These high scores were generally maintained at posttraining and at follow-up.

All children showed improvements on Selection 2 (cognitive flexibility). Child 1 made progressive improvements on Selection 2, from 55% at baseline to 73% at posttraining and 100% at follow-up. Child 2 doubled her baseline score of 50% and maintained 100% at follow-up. Child 3 scored 83% at baseline. He showed improvement to 92% at posttraining and maintained this at follow-up. Child 4 more than tripled his baseline score from 33% to 100% and maintained the high score at follow-up. 

### 3.3. Control Test: Attention Sustained

Table 3 shows the scaled scores for the children's performance on the Attention Sustained subtest. Scaled scores for Correct Responses and Error Responses are shown separately.

Child 1, Child 2, and Child 4 showed no changes in performance on the scaled scores for both correct responses and error responses. Child 3 showed an increase of 6 points on scaled correct responses from baseline to posttraining, and a decrease in 1 point in scaled error responses at posttraining. 

### 3.4. Final Feedback Questionnaire

The parents of Child 1 and Child 4 reported no changes on any of the items on the Final Feedback Questionnaire. Both Child 2's mother and Child 3's mother reported changes in the category of *appropriate language and communication in social contexts*. Child 2's mother reported that Child 2 “seems to initiate social interaction with more appropriate language (e.g., Hi. What's your name? I like your hair style rather than … what colour is your nail polish?).” Child 3's mother reported that Child 3 “can answer question(s) appropriately” and that since the beginning of the study, Child 3 “is more flexible and like(s) to try something new.”

## 4. Discussion

Overall, three of the four children showed 100% nonoverlapping data on the VR-test. The fourth child showed 60% nonoverlapping data. No changes were found on the control test for three children, while the fourth child showed significant improvements on this test. Finally, parents of two children reported changes in the presence of appropriate social behaviours.

The primary objective of the study was to explore if the novel VR-CR intervention could improve contextual processing in children with autism. According to the standards set by Logan and colleagues [34], the highest level of evidence for single-subject research designs (Level 1) can be achieved by “concurrent or non-concurrent multiple baseline designs (MBD) with clear-cut results; generalizability if the MBD design consists of a minimum of three subjects, behaviours or settings.” In determining “clear-cut results,” Scruggs and Mastropieri [33] assert that a treatment outcome with over 90% nonoverlapping data can be considered a “highly effective treatment.” The results of this study demonstrate clear 100% nonoverlapping data for three children with three different, nonconcurrent baseline lengths. Thus, the current study fulfills the criteria for a Level 1 single-subject design study.

Changes in abstraction and cognitive flexibility were also measured to verify improvements in contextual processing. As expected, initial performance on Selection 2 (cognitive flexibility) was low. Three children performed at chance (50%) or below. After the training phase, all children displayed substantial improvements and maintained a high level of performance at follow-up. Both Child 2 and Child 4 showed notable improvements on Selection 2 on the FIST-m, doubling and tripling their scores, respectively. Child 3 also showed improvements; however, his performance on Selection 2 at baseline was initially high. Although there is no normative data available for comparison, studies performed on the original FIST showed that during a crucial phase of cognitive flexibility development in typical children (between 4 and 5 years), 5-year-olds performed almost 18% better than 4-year-olds on Selection 2 [27]. The difference reflects the rapid development in cognitive flexibility within that one-year span. Three children, Child 1, Child 2, and Child 4, matched or exceeded this spike in development within only two weeks. Thus, this is emerging evidence that cognitive flexibility can be improved in this subgroup of children.

Although impairments in abstraction have been demonstrated consistently in the literature [16], the children in the current study demonstrated unexpectedly high abstraction performance (FIST-m, Selection 1) at baseline. Findings from Ropar and Peebles [16] indicate that autistic children have difficulty accessing “high-level” abstract categories such as sports or games. Although the current VR-CR intervention required the children to abstract qualities *above* the salient or perceptual, the demands of the current task were demonstrated to be within the children's range of capabilities.

The control test was used to verify that the VR-CR intervention could specifically target contextual processing of objects rather than overall cognitive functioning. On the Attention Sustained Subtest, three of the four children showed no changes over the course of the study, supporting the specificity of the intervention. However, one child, Child 3, showed significant improvements on one component of the subtest from pretraining to posttraining. Child 3 improved by 6 points on the correct responses score, which resulted in a 10–13% increase in his ranked percentile. This change is significant and may have been influenced by a couple of factors, including maturational effects and the level of rapport within the child-instructor therapeutic relationship. Overall, based on the results of the control test, the VR-CR intervention demonstrated specificity to contextual processing for three of four of the children. 

The final objective of the pilot study was to explore changes in context-related behaviours that may have occurred during the course of the study. The comments obtained by parents on the Final Feedback Questionnaire were mixed. Child 1 and Child 4's parents reported no behavioural changes that occurred between the start and completion of the study. Child 2 and Child 3's mothers reported changes in the category of *appropriate language and communication in social contexts*. However, it is noted that both Child 2 and Child 3 were actively engaged in weekly social activities such as swimming and horseback riding, while Child 1 and Child 4 were not. Thus, the positive behavioural changes reported are likely due to factors outside of the intervention itself.

The major limitation of this study is the high baseline levels achieved by all the children prior to intervention. This suggests that the participating children were already showing high levels of contextual processing ability before the study, particularly in abstraction. Assessing performance on the VR-test prior to enrolment would help to exclude children from the study who are already performing at high levels on the task. In addition, determining language levels or verbal intelligence at baseline may help to clarify those children who would benefit maximally from this type of cognitive intervention.

A second limitation of this study is the lack of multiple, independent assessors. The degree of therapeutic effectiveness and test performance was likely influenced by the development of rapport between the researcher and each child. To minimize this effect of researcher bias in the current study, there were no subjective rating scales involved. In addition, scoring of the VR test was verified through computer records. 

## 5. Conclusion

This pilot study evaluated a novel intervention that combined virtual reality technology with traditional cognitive rehabilitation methods to address impairments in *contextual processing of objects* in children with autism. All children who participated in the VR-CR program demonstrated statistical improvements in overall contextual processing ability and improvements in cognitive flexibility. However, a larger-scale study will need to evaluate the magnitude of change resulting from the intervention and the degree that cognitive improvements generalize to behaviour changes.

Overall, this pilot study provides initial supporting evidence for the efficacy of this particular VR-CR intervention. Continued research may further generate support for the broader use of the VR-CR approach with children with autism. The hope is that comprehensive interventions will be developed to target *both* cognitive and behavioural levels of impairment in children with autism. This may lead to greater overall improvements in the daily functioning and quality of life of these children.

## Figures and Tables

**Table 1:** Participant demographic information.

	Child 1	Child 2	Child 3	Child 4
Gender	Boy	Girl	Boy	Boy
Age	6 years, 7 months	8 years, 11 months	6 years, 1 month	7 years, 11 months
Grade	1	4	1	2
Diagnosis	Autism	Autism	Autism	PDD-NOS
CARS score	32.5	32.5	30	33
Nonverbal IQ	98	111	139	119
Siblings	Brother (9 years, 5 months)	Sister (6 years, 1 month)	None	Sister (9 years, 11 months)
Mother's education level	Bachelor degree	Doctoral degree	Bachelor degree	Bachelor degree
Father's education level	Postgraduate degree	Bachelor degree	Bachelor degree	Bachelor degree
Primary language spoken at home	English	Mandarin Chinese	Mandarin Chinese	English
Extracurricular activities	Weekly 1 : 1 tutoring for academic subjects	Weekly swimming class, cooking class, and therapeutic horseback riding	Weekly social skills group for children with autism	None
Baseline length	3 sessions	4 sessions	5 sessions	6 sessions

Abbreviations: CARS: Childhood Autism Rating Scale; [25], ASD: Autism Spectrum Disorder; PDD-NOS: Pervasive Developmental Disorder, not otherwise specified. Note: nonverbal IQ scores are derived from the Brief-IQ screener from the Leiter International Performance Scale [26].

**Table 2:** Percentage accuracy scores for Selections 1 and 2 of the FIST-m at pretraining, posttraining, and follow-up for each child.

	Selection 1	Selection 2
Child 1		
Pretraining	92	55
Posttraining	92	73
Follow-up	75	100
Child 2		
Pretraining	100	50
Posttraining	100	100
Follow-up	92	100
Child 3		
Pretraining	100	83
Posttraining	100	92
Follow-up	100	92
Child 4		
Pretraining	100	33
Posttraining	92	100
Follow-up	100	100

**Table 3:** Scaled scores for correct responses and error responses of the attention sustained subtest at pretraining, posttraining, and follow-up for each child.

	Scaled scores of correct responses	Scaled scores of error responses
Child 1		
Pretraining	1	10
Posttraining	1	10
Follow-up	1	10
Child 2		
Pretraining	1	12
Posttraining	1	12
Follow-up	1	12
Child 3		
Pretraining	10	8
Posttraining	16	7
Follow-up	16	7
Child 4		
Pretraining	1	3
Posttraining	1	3
Follow-up	1	3
